# Selenium Status of Southern Africa

**DOI:** 10.3390/nu16070975

**Published:** 2024-03-27

**Authors:** Pompido Chilala, Sylvie Skalickova, Pavel Horky

**Affiliations:** Department of Animal Nutrition and Forage Production, Faculty of AgriSciences, Mendel University in Brno, Zemedelska 3, 613 00 Brno, Czech Republic; pompido.chilala@mendelu.cz (P.C.); sylvie.skalickova@mendelu.cz (S.S.)

**Keywords:** selenium, feed, food, nutrition, livestock, humans, biofortification, Southern Africa

## Abstract

Selenium is an essential trace element that exists in inorganic forms (selenite and selenates) and organic forms (selenoamino acids, seleno peptides, and selenoproteins). Selenium is known to aid in the function of the immune system for populations where human immunodeficiency virus (HIV) is endemic, as studies suggest that a lack of selenium is associated with a higher risk of mortality among those with HIV. In a recent study conducted in Zambia, adults had a median plasma selenium concentration of 0.27 μmol/L (IQR 0.14–0.43). Concentrations consistent with deficiency (<0.63 μmol/L) were found in 83% of adults. With these results, it can be clearly seen that selenium levels in Southern Africa should be investigated to ensure the good health of both livestock and humans. The recommended selenium dietary requirement of most domesticated livestock is 0.3 mg Se/kg, and in humans above 19 years, anRDA (recommended daily allowance) of 55 mcg Se/per dayisis recommended, but most of the research findings of Southern African countries have recorded low levels. With research findings showing alarming low levels of selenium in soils, humans, and raw feed materials in Southern Africa, further research will be vital in answering questions on how best to improve the selenium status of Southern African soils and plants for livestock and humans to attain sufficient quantities.

## 1. Introduction

Selenium was officially recognized worldwide to be of nutritional value in 1957 due to its vital role in growth, fertility, and antibody production in animals [1,2]. Quantities of one or more selenoproteins (glutathione peroxidase and selenoproteins) are used as a measure of selenium status in livestock and humans, with plants being the main source of selenium for both livestock and humans [3,4]. Plants take in selenium from the soil at an optimum pH, mostly alkaline and in the form of inorganic compounds that easily dissolve in water. Selenium present in the soil is transported into the plant by sulfate transporters present in the plasma membrane of root cells [3]. Selenium exists in many food and plant products, naturally as a trace element and is also added as a supplement; the two main forms in which selenium exists are inorganic (selenate and selenite) and organic (selenomethionine and selenocysteine). In general, Southern Africa has been known to have very low concentrations of selenium in plants, leading to deficiencies in humans and livestock [5,6]. However, information on the selenium status of animals, soil, plants, feed, and human populations of Southern African countries is limited.

Why is Selenium important to Southern Africa?

Selenium is a vital element of many enzymes and proteins; these proteins are called selenoproteins, which play a big role in making DNA and preventing cell damage and infections from pathogens [7,8]. Selenoproteins are actively involved in the metabolism of thyroid hormones and reproduction [9,10,11,12]. Selenium has also been documented to aid in the normal immune function of animals, and further studies on whether higher levels of selenium can stimulate the immune system are still ongoing [13,14]. Selenium deficiencies in humans were discovered to be among the major causes of problems in reproduction and growth, and slight deficiencies are well known to cause myodegenerative diseases (weakness of muscles) and depressed mood, confusion, and anxiety in humans [14,15,16].

Some studies on the relationship between selenium and cancer are still a hotly debated issue in human health and other fields of science [8,17]. According to research findings by [8], there is a strong indication that selenium reduces the risk of cancer due to the role it plays in the immune system. However, further research using randomized control trials indicated that cancer risk is not reduced by selenium supplementation but may instead increase the risk in some types of cancer (skin and prostate cancer) [17]. Further findings by [18] strongly indicate that selenium supplementation increases the risk of diabetes.

According to research, HIV infections have been linked to selenium deficiencies, which are associated with a high risk of death due to tuberculosis and diarrheal diseases [19,20]. With these results, many health institutions in Africa recommend selenium supplementation in HIV patients [14,21,22,23,24]. In some trials, selenium supplementation in HIV patients had reduced hospitalization and improved CD4 counts and diarrheal morbidity; however, the evidence is not very clear and will require further investigation [19]. Other findings from a randomized control trial in Rwanda indicate that selenium supplementation for 2 years significantly reduces the decline of the CD4 cell count among patients under antiretroviral treatment [22]. Further research findings on autoimmune disease concluded that a dose of selenium contributes to the management of complications of autoimmune diseases and aids in patients’ survival, and this may be attributed to the anti-inflammatory effects of selenium [25]. With research conducted around the world, the results clearly indicate that selenium supplementation or inclusion in the diet of livestock can enhance the economic performance of livestock farms and improve human health in Africa. In this review, we aim to combine established and novel knowledge on the selenium status of countries in Southern Africa and possible ways to increase these levels based on the available research findings.

## 2. Importance of Selenium and the Effects of Its Deficiency

### 2.1. Soil

Concentrations of selenium in soils across the world differ, mainly as a result of varying environmental conditions and the parent structure of soil [3,26,27,28]. Selenium mainly comes from soil erosion, which accounts for between 40 and 50% of selenium that exists naturally on Earth [29]. Selenium biogeochemical cycling in an ecosystem is the main basis for understanding selenium characteristics in microbial systems, soil, and plants [30,31]. The levels of selenium in soils are mainly linked to plant selenium concentrations, and its availability in the soil varies depending on the texture, organic matter, type of soil, and amounts of rainfall in a region [3,12,32,33]. The presence of selenium in soil aids in the prevention of damage induced by climate change, mainly extreme temperatures, drought, and salinity of soils. All crops are known to accumulate selenium from the soil in inorganic forms, namely selenates and selenites, hence making soil an important source of selenium for plants and animals [12,34]. According to research conducted by [35] on rice, low concentrations of selenium are beneficial to plants, but the range between optimal and toxic is very narrow [35]. There is a huge challenge for people to obtain the required minimum selenium concentrations in agricultural produce due to soils having inadequate selenium to pass to plants, as seen in the findings [36] on challenges with selenium deficiency in northeast China, where areas with increased agricultural production are the most affected [36]. According to various research, the analysis of soil samples for determining deficiencies of selenium in farm animals is not recommended due to the fact that soil selenium contents include elemental selenium and selenites, which are inorganic and cannot be utilized by plants [27].

### 2.2. Pasture and Crops

According to [37], the understanding of how selenium is accumulated by crops in the soil is limited despite being extensively studied together with elements such as sulfur and arsenic. Selenium is obtained by plants at different levels depending on the plant species, amount of rainfall, and type of fertilizer application method used [38]. Selenium is not among the main essential nutrients required by plants, although it aids in growth and survival in certain environments [39]. The beneficial aspects of selenium to agricultural crops range from the transport of different solutes and metabolites within cell organelles, chloroplast, and plastid membrane stability to reduced cell viability and the regulation of membrane structures [12,34]. Adequate selenium in crops has also been observed to enhance photosynthesis-induced plant yields in plants with decreased oxidative stress and delayed senescence [34]. In required concentrations by crops, selenium aids in protecting plants from stressors, reduced growth, and pathogens [40]. According to [36], low doses of selenium can improve plant productivity or the phytoremediation parameters of plants by improving photosynthesis and enhancing the plant’s capabilities to tolerate stress. According to [41], fertilization with selenium on wheat did not affect the grain yield of the crop, but an increase in the selenium content of the grains was recorded. Research results by [41] further indicated that the accumulation of selenium by plants does not depend on the dose of the selenium fertilizer but on the phases of plant growth when selenium is introduced. Research done by [38,39] on guinea grass and star grass indicated conflicting results, proving that the uptake of selenium can also be dependent on the species. Based on further research on 20 seleniferous plants, mainly Astragalus species (which is not palatable to livestock), have been identified as selenium accumulators among plants [42]. Other research findings indicated that selenium concentrations were higher in green grass than in leguminous plants [42,43]. The main benefit of having adequate selenium in pasture and food crops is mainly to transfer it to animals that require selenium for normal biological functions of their bodies [44].

### 2.3. Farm Animals

The addition of selenium to animal feed as a supplement is mainly associated with health coupled with immunomodulatory activities and oxidative damage protection [44,45]. Selenium is taken up by animals when feeding from pastures fertilized by selenium, drenches, injections, selenized lick blocks, premixes, and intra-ruminal selenium pellets bought in shops [11,45]. In pigs and other livestock, selenium supplementation to the diet is essential for improving their immune function, growth, and meat quality parameters [4,46]. In ruminants, a number of research findings have classified selenium as an essential trace element required for normal growth and fertility and for preventing health disorders such as mastitis [7,47]. The findings suggest that selenium is very important in maintaining a healthy immune system and in helping fight against diseases in combination with vitamin E [47,48,49]. According to [50], animals deficient in selenium have problems with milk production, fertility, and mastitis, as well as premature, weak animals at birth and with abortions [11,47,49]. In other research, young animals with severe deficiencies in selenium exhibited poor growth, chronic diarrhea, and nutritional muscular dystrophy; nutritional muscular dystrophy was prevalent in young calves [44,47]. According to [47], cattle feed supplementation with selenium as an essential element was an effective way of enhancing dietary exposure through commonly consumed foodstuffs derived from dairy. For many decades, milk has been considered to be a primary source of selenium for human diets; concentrations of selenium in milk are mainly thought to be controlled by the source and quality of fodder given to lactating cows [45,47]. However, research findings by [50] found no relationship between the fodder and selenium contents in milk. Livestock consume selenium from the plants they eat, and as a result, plants are able to provide the organic form of selenium to animals. Selenium in an animal’s body is mainly stored in the liver with continuous supply, enabling the best possible production by animals [44,48]. Selenium is known to be toxic to animals if large doses are consumed [51]. However, animals have a high need for selenium compared to plants due to the active role it plays in the function of the immune system [11,52]. In livestock production, assessing the selenium levels in total mixed rations for animals is done as part of a herd health program [1].

### 2.4. Human

**Figure 1:**
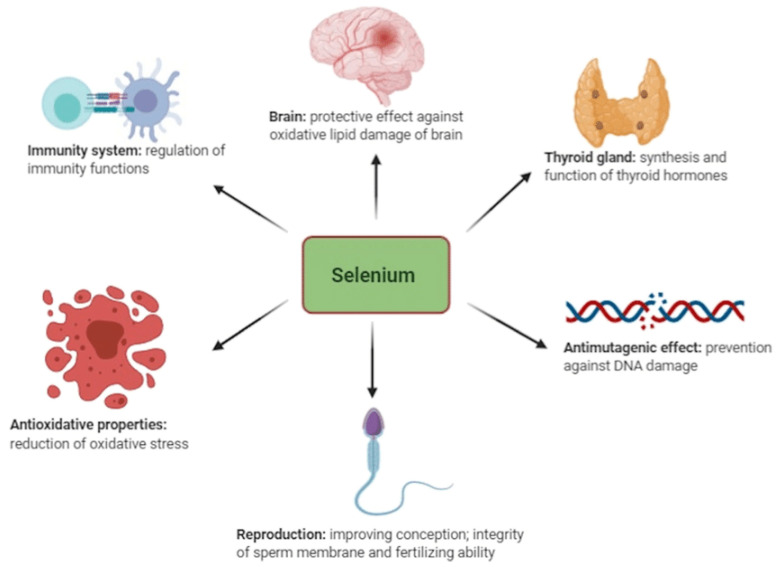
Functions of selenium in animals adapted from Kieliszek et al. [30].

Among the total amount of selenium in an average human (~3–20 mg), around 47% is found in skeletal muscles/fiber cells and about 4% in the kidneys [53]. Generally, the presence of selenium is determined by its concentration in blood serum [7,54,55]. For maintaining homeostasis, the human body cells require balanced nutrition and adequate micronutrients from trace elements [10,30,56]. Selenium is known to be part of the required micronutrients among the trace minerals utilized by the human body; others are antioxidants and vitamins, which are vital for performing various regenerative processes in the human body, such as immunity development for fighting pathogens and managing oxidative stress [10,32,51] (Figure 1).

Many researchers have found selenium to play a vital role in the pathogenesis and pathophysiology of several disorders in humans, mainly linked to its antioxidant properties such as cancer, HIV, thyroid issues, oxidative stress, reproductive disorders, inflammations, and other immune responses [8,57,58]. In humans, it is important to address selenium deficiencies, as they are crucial for mitigating autoimmune diseases and improving health outcomes, particularly in vulnerable populations like pregnant women—especially in individuals recovering from severe illness or COVID-19 [20,59]. Implementing population-wide measures like that of Finland’s selenium fortification program can provide significant public health benefits and combat the often-overlooked risks of selenium deficiencies in Africa.

With selenium being part of the selenoproteins in the immune system, selenium poses enzymic roles and structures that are used as antioxidants and catalysts for producing active hormones for the thyroid gland [59,60]. According to the findings by [24], selenium has been noted as a key nutrient required for impeding the occurrence of virulence and reducing HIV advancement to AIDS in humans [22]. Based on the findings by [61], selenium is crucial in the human body during viral infections such as COVID-19 by assisting in redox homeostasis, reducing oxidative stress, and providing an antioxidant defense. In other research findings, selenium was proven to be required for reducing sperm motility and the risk of miscarriage in women [9]. In other studies, increased selenium intake by humans has been associated with reduced cancer risks, and further research is being conducted to prove or refute this hypothesis [8,17,36,62]. In this regard, selenium is seen to have various health benefits, but a low or diminishing selenium status in some parts of the world has been noticed, with some African, Asian, and European countries showing signs of great concern to health organizations [15,18,55,63]. Lack of selenium has been linked to many diseases due to reduced functions of glutathione peroxidase, coma, sudden death syndrome for infants, asthma, and irregular heartbeats [16,54]. Keshan disease is one of the diseases that is directly linked to a lack of selenium in humans [64,65]. Keshan disease is a regularly occurring highly lethal cardiomyopathy that was first reported in 1935 in northeast China, in a county called Keshan [30,31,36,64]. The main clinical signs of Keshan disease include acute failure and cardiac arrhythmia. On another hand, excess selenium intake in human diets has been linked to causing food poisoning with complications such as diarrhea, vomiting, and nausea [10,11,62]. Selenium toxicity occurs with some acute signs, which include fatigue, hair loss, irritability, nausea, nail discoloration, foul breath, and vomiting [10,66]. Other symptoms recorded include tremors, heart attack, difficulty breathing, heart failure, and kidney failure [10].

## 3. Current Knowledge of Selenium Status in Southern Africa

### 3.1. Soil

Soil is crucial in providing selenium to plants, resulting in the accumulation and generation of bioavailable selenium for humans and animals [37,67,68,69]. Selenium in the soil is dependent on the geological parent material, being the main contributing factor [3,15,27,37,40]. According to [6], the arenosols of eight Southern African countries were estimated to be 168.6 million ha, with Angola having the largest share (63.4 million ha) and Botswana covering 51% of the total land area. Arenosols, which are mostly sandy soils with a pH of less than 6, cover vast areas of Namibia, Mozambique, South Africa, and Angola. Based on research in Lithuania, sandy soils were found to have lower selenium contents compared to organic and calcareous soils, making them deficient most of the time [37]. According to the world soil reference base by FAO [28,70], arenosols are sandy soils with little or no development and are unconsolidated, with typically translocated sandy parent material. The results of [31] are in tandem with the findings by [71] in Malawi, where the selenium content in maize was higher in calcareous soils with an alkaline pH than other soil types. According to [72], most soils have a selenium content ranging from 0.1 to 10 μg/g; however, other soils contain higher amounts of up to 8000 mg kg^−1^ in seleniferous soils. In selenium-deficient areas, the selenium content may range from 0.005 to 2 μg/g [72].

In mining, selenium is a by-product of mineral extraction processes and is primarily obtained from the anode mud of refineries for copper and other minerals [73]. Based on the research findings by [74], mining is a prime contributor to hazardous selenium dumping in the water systems and brings about acute and chronic impacts on living organisms. Copper is mined in many Southern African countries, with the main copper producers being Zambia, Botswana, Namibia, South Africa, and Zimbabwe. According to [75], samples collected from a mining tailing dam in the UK of both natural and waste deposits exhibited elevated trace element concentrations of selenium and other elements; the concentrations were of both economic and environmental consideration. The findings by [75] are important for investigating the levels of pollution caused by selenium waste to water and the effects on humans and livestock in the surrounding areas.

Marginal to acute selenium deficiencies in farmlands have been reported to occur in many regions of Africa, with a particular emphasis on Southern Africa [5,15,76,77]. Research findings show that the only two countries in Southern Africa to report their selenium status using a nationally representative survey are Malawi and South Africa [12,78]. A decline in the selenium content of the soil, as a result of climate and soil interactions, was seen to be prevalent in agricultural production fields in most regions of Southern Africa [6,76]. The soil pH was observed to play a major role in the availability of selenium to field crops in Southern Africa; soils with a low pH tend to have less selenium available to plants [3].

It was further observed that selenium is accessible by plants in soils with a pH above 6.5 [32]. Based on [33]’s research findings, soils containing less than 0.05 µg Se/g are classified as deficient, and those that contain more than 5 µg Se/g are considered to be sufficient and can be called seleniferous soils. Based on the available data from 12 countries of Southern Africa, only two countries, namely Malawi and South Africa, have undertaken soil tests to ascertain the status of selenium in soils. According to the geostatistical modeling results, various results indicate that selenium deficiency risks are mainly influenced by the soil types in Malawi and the rest of Southern Africa [5,33].

### 3.2. Field Crops and Pastures

Plant-based nutrients are known to be the major dietary sources of selenium and other trace elements in many parts of the world; meat and pharmaceutical supplements are the other sources [48,49]. It is observed that the human selenium accumulation status of a population and levels are highly dependent on the levels of selenium in the soils [27,79]. Soil concentrations of selenium are manifested in the crops produced for human and animal consumption; this relationship between soil and plants determines the selenium status of that region [26,79,80]. Selenium is translocated efficiently by plants from the soil, and leaf contents of selenium are observed to be a good proxy for the relative grain selenium concentration and other plant parts [26,42,67,70]. According to [5], out of all the maize samples that were analyzed in South Africa, 94% of maize contained below 50 µg selenium/kg and can thus be classified as deficient from a livestock and human nutritional point of view. This research clearly showed that the maize grain of South Africa proves to be a poor source of selenium for livestock and humans [77]. In other experiments, it was observed that a stronger positive correlation exists between the grain selenium concentration of field crops, such as maize, wheat, soya beans, and sunflower, with a pH above 6.5 [33]. In Malawi, selenium concentrations in crops were greater in calcareous than non-calcareous soils [33]. According to [81,82], field crop products that possess more than 0.1 mg Se kg^−1^ protect livestock from selenium deficiency disorders and related diseases. Feeding rations from crops with inadequate selenium for animals will cause white muscle disease in calves, goats, and sheep [44]. In poultry, exudative diathesis has been reported, and Mulberry heart disease in swine is due to deficiencies in the feed rations from crops [45,83]. In humans, selenium deficiencies have been linked to heart disease, cancer, and other life-threatening conditions [18,23]. Research suggests that selenium deficiencies can be prevented by biofortification using transgenic, conventional, or agronomic methods [78,84,85]. Selenium injections are recommended for humans and livestock, with a greater emphasis on females in their last stage of gestation and young stock shortly after birth for normal growth [47,86]. Food and feed additives for both humans and livestock have proven to be effective methods in improving selenium levels in the body [49].

### 3.3. Content of Selenium in Pasture

Even though literature exists on the selenium status for grazing animals of South Africa, mapping has not been done to give a clear understanding of the situation at hand [5]. With limited research findings, the available data indicate that vegetation in the arid regions of Southern Africa provides enough selenium to be sufficient to meet the requirements of grazing herbivores, while other results indicate deficiencies at alarming levels; this can be attributed to soil types and the soil pH in different regions [12,33,71]. In most grasslands of Southern Africa, little or no information on the status and availability of selenium for grazing animals exists to compare with [12]. Selenium was observed in drinking water for ruminants at levels that are not sufficient to meet the dietary requirements of animals in South Africa [77]. Previous studies have revealed that approximately 90% of the samples from pastures in Southern Africa are below the 0.1 ppm limit, with the average being below 0.04 ppm. Cattle have a guide of having 0.1 mg Se/g in blood and would fail to maintain these levels if grasses are low in selenium. With the coming of silvopastoral systems of agroforestry in Southern Africa, tree leaves and other parts are included in livestock feeding as a result of the observed high levels of nutrients beneficial to animal nutrition [87]. Research shows that Moringa oleifera has an exceptional ability to absorb and accumulate minerals, such as selenium and sulfur, even in soils poor in minerals [87]. Deficiencies of selenium, sulfur amino acids, and vitamin A are common in crop produce, especially in Sub-Saharan Africa, and this has important health and medical implications for livestock and humans [87].

### 3.4. Content of Selenium in Protein Feeds

The most common and proven effective way of maintaining levels of selenium and improving them in humans can be acquired through introducing premixes containing biologically available selenium forms into animal feeds [88]. According to [11], a selenium addition to animal feeds in research conducted in Serbia ensured high levels of selenium in eggs, milk, and meat. Selenium sourced from animal meats is safe for human consumption since homeostatic control is highly developed in all vertebrate species [89]. The use of sodium selenite as a source of selenium in animal nutrition is practiced by many compound feed producers in Southern Africa, provided that the total maximum authorized content of selenium in a complete feed is respected [1]. A survey of veterinarians practicing in the beef industry of South Africa and Namibia revealed that selenium and phosphorus are the most prescribed minerals due to selenium bioavailability in the cattle diet [77]. It has become standard practice in most parts of Southern Africa to include selenium in dairy cattle diets. Addition rates of 0.3 mg Se/kg in animal feeding are recommended by many countries due to the low toxicity and proven beneficial results [45]. Increased selenium contents in pork provided results of low toxicity and opportunities for using organically sourced selenium at high does to enhance the selenium content in pork without affecting any production parameters [45]. In cattle, beef has been regarded as the main source of selenium for humans; therefore, feed additives would boost the selenium status in cattle and people who consume the meat. According to [60], an increase in selenium in the diet was observed to enhance glutathione peroxidase activities and improve fertility in male animals.

### 3.5. Selenium in Livestock

Selenium has been proven to be a very important trace element in the nutrition of dairy cattle, according to research that focused on the role of antioxidant defense and selenium [44,47]. The recommended daily requirements for cattle are estimated to be 100 μg/kg DM (dry matter) for beef cattle and 300 μg/kg DM for dairy cows due to being milk-producing animals [52]. According to [52], selenium use in cattle diets is negatively affected by feed rations high in fermentable carbohydrates, sulfates, nitrates, calcium, and hydrogen cyanide. Research strongly suggests that selenium supplementation may decrease the occurrence of metritis and ovarian cysts in postpartum periods [52]. Research findings by [9] strongly suggest that fertility is increased when selenium is added to the diet due to reduced embryonic death in the first months of gestation. With the introduction of organic selenium in feed, farmers and scientists noticed a better transfer of selenium in calves compared to supplementation with inorganic selenium [49]. The other additive to feed is selenium yeast, which has proven to significantly increase the contents of selenium in animal feed and the percentage of polyunsaturated fatty acids in milk as opposed to sodium selenite addition [90]. In the swine diet, selenium is included as a trace mineral in premixes and is a component of the glutathione peroxidase enzyme that protects membranes at subcellular and cellular levels from lipid peroxide damage [91]. Selenium was also found to play a vital role in the metabolism of thyroid hormones in controlling metabolism coordination [48]. It is important to note that selenium is a unique element that is toxic at slightly higher concentrations than required for the normal metabolism of an animal [92]. The recommended dietary requirements for swine range from 0.3 mg/kg in piglets to 0.15 mg/kg in finishers, sows, gilts, and boars [7,93]. Research shows that using selenium supplements higher than the recommended dose can be easily toxic, even when feeding as little as 5 to 10 mg/kg in the diet [45]. The main signs of selenium toxicity in swine that were recorded are hair loss, liver and kidney damage, edema, hoof loss, and disorders of the nervous system [56]. In poultry, the results have proven that eggshell-quality breaking strength is improved when selenium is added to the diet of laying hens [94,95]. In trials conducted on broilers, organic selenium in the diet amplified selenium transfer to the muscles and the build-up of selenium reserves in the body; this resulted in chickens that had improved resistance to stress and a positive effect on their immunity [96]. The other parameters that increased were meat quality and gut health.

### 3.6. Selenium in the Human Population

Selenium is a very important micronutrient required for the normal functioning of some enzymes and proteins in the body [16]. Selenium deficiencies were marked as a health problem of concern for 0.5 to 1 billion people worldwide based on the proven benefits of improving the physiological functions of the human body [81].

The main antioxidant enzyme that depends on selenium is glutathione peroxidases, which is essential in preventing lipid peroxidation and maintaining intracellular homeostasis, as well as redox balance and iodothyronine deiodinases, which are important mediators of thyroid hormone actions [97]. Selenocysteine is the most common form of selenium in the body of an animal and aids in the function of selenoproteins [59,97]. Selenium deficiencies have been observed in several countries throughout the world, especially in African countries, with the main attribute being low-income levels, creating a barrier to afford a diet that is rich in selenium and soils being poor in selenium [21]. Research in Malawi suggested that selenium status in the human population is mainly dependent on local soils and agriculture; further findings from Zambia, Zimbabwe, and South Africa also agree with the findings in Malawi [54,78,98]. Selenium is well known to have a low window from being therapeutic to toxic [29]. Available information strongly indicates that Keshan disease, Kashin–Beck disease, cardiomyopathy, and osteochondropathy are the main diseases that arise from selenium deficiency [16]. In new studies, selenium deficiencies have been linked to other conditions, such as decreased immune function, increased viral virulence, and thyroid autoimmune disease, with Southern Africa battling with high numbers of people with immune deficiencies [23]. Selenium in the immune system is known to aid in functions of the immune system for populations where human immunodeficiency virus (HIV) is endemic, as studies suggest that a lack of selenium is associated with a higher risk of mortality with those who have HIV [14,19]. According to the recent research conducted in Zambia on the status of selenium in the blood plasma of a sample population, a total of 660 plasma samples from 391 adults and 269 children were analyzed. Adults had a median plasma selenium concentration of 0.27 μmol/L (IQR 0.14–0.43) [21]. Concentrations consistent with deficiency (<0.63 μmol/L) were found in 83% of the adults. Among the children, 24% had plasma selenium less than 0.41 μmol/L [13]. Similar separate research conducted in Zimbabwe, Mozambique, and Malawi strongly suggests that selenium deficiency is widespread in Southern Africa and could, in part, be related to the socio-economic status of the people [12,21,54]. Supplementation and selenium-rich fertilizers are needed to boost levels in the maize, which is a staple food in most Southern African countries.

## 4. Crop Biofortification Strategies as a Tool to Improve Selenium Levels in Southern Africa

Biofortification was proven to be a useful tool for the addition of selenium in food crops with promising outcomes and it is achievable and economical to combat selenium deficiencies in humans and livestock [34,53]. Biofortification involves the addition of essential micronutrients and any other health-promoting compounds to agricultural crops or pasture, resulting in improved nutritional diets and quality consumed by livestock and humans [32]. According to [34], biofortification is an easy, affordable, innovative, and cost-effective method of handling micronutrient deficiencies in livestock and humans. However, outcomes of biofortification are highly dependent on the environmental and economic characteristics of local soils and food systems, with particular emphasis on farmers adopting the innovation and the population consuming the produce [15,32].

### 4.1. Soil

Research on grain crops showed that sodium selenate and sodium selenite application through biofortification to the soil has beneficial effects of reducing heavy metal concentrations in grains and husks [68]. In other findings, biofortification with selenium in the soil was noticed to inhibit the transportation and accumulation of mercury in plants, while further research is required to understand this interaction fully [32]. According to [99], for farmers to economically and efficiently implement biofortification measures, he recommended the utilization of the regression models to precisely predict the availability of selenium in the soil and further discovered that the retention of selenium in the soil is usually a multifaceted process, mainly characterized by not only the surface charges of the soil but by anions such as sulfates, phosphates, and nitrates present in the soil due to the displacement of selenium in the adsorption complex of the soil. Most European countries were characterized by low levels of selenium in the soil, with the Balkans region having low concentrations of between 0.024 and 0.45 µg Se g^−1^, while normal levels of soil selenium range from 0.1 to 2.0 µg g^−1^ [99]. According to [26], toxicity by selenium in the soil is exerted between 30 and 324 µg g^−1^, with healthy soils containing an average 2 µg g^−1^. Through biofortification by selenium fertilization, breeding coupled with genetic manipulation of crops was discovered to be the most effective and safest measure at improving selenium levels in humans and animals, mainly due to the dietary intake of selenium being the most practical pathway of providing sufficient selenium supplies to humans and animals [32,100]. Soil amendments through conventional or assisted breeding can be achieved by enhancing soil quality through developing plant varieties using traditional or advanced breeding techniques. The process involves selecting or genetically modifying plants to improve their ability to absorb and accumulate selenium from the soil [2,100]. By breeding plants with higher selenium uptake capacities, the overall selenium content in soils can be increased, addressing potential deficiencies and improving soil fertility [2]. Thus, breeding plays a crucial role in augmenting the soil’s selenium levels, contributing to agricultural productivity, and addressing nutritional concerns [32,80,85,101]. Many research outcomes in Europe on how they managed to increase the selenium status of their populations have determined that biofortification was the most effective method for increasing selenium concentrations in most widely cultivated soils with cereals, such as wheat, barley, and maize [2,55,100,102]. With Southern Africa facing similar challenges as Europe did in previous years, it is advisable to use the same approach used by European countries of biofortification to increase the selenium levels in feed and foodstuffs.

### 4.2. Pasture and Crops

The biofortification of field crops and pastures with selenium can be achieved using three methods, namely, transgenic, conventional, and agronomic methods [101]. Transgenic biofortification mainly incorporates genetic tools utilizing genetic engineering and plant breeding [2,53]. Conventional biofortification involves the use of biotechnology techniques to enhance the micronutrient status of a crop [32]. Agronomic biofortification encompasses soil amendments through conventional or assisted breeding, which can be achieved through conventional selenate or selenite fertilization, and nanosized biofortification is achieved by using SeNPs applied to the leaves or soil [34,101]. Ref. [103] stressed that improving the micronutrient contents of staple foods through biofortification can be deemed a cheap and cost-effective agriculture-based intervention to reduce the health burden of micronutrient malnutrition in people and livestock. The findings of [103] through the DALY (disability-adjusted life years) framework show that out of the 10.6 million DALYs lost every year in China from micronutrient malnutrition, approximately 1.2 million and 4.9 million DALYs would be rescued if multi-biofortified rice was introduced. The authors of [103] further state that previous biofortification studies were criticized by some scholars based on their focus on single micronutrient deficiencies—a problem that multi-biofortification resolves—and due to the fact that micronutrient deficiencies are known to occur simultaneously, it makes multi-biofortification the preferred option [103,104]. Enrichment of crops with selenium through biofortification is proven to promote higher antioxidant activities of grains and increased nutrients [85,105].

According to trials that involved the biofortification of maize in Malawi [78], the results strongly suggest that selenium levels of maize improved with biofortification. Research outcomes by [85] obtained in Kenya suggest that the selenium concentration increases on average by 3 µg/kg^−1^ in maize and by 10 µg/kg^−1^ in beans for each gram of selenium applied as sodium selenate to the soil. Foliar selenium application to crops was found to be more effective and increases the selenium concentration in grains on average by 18 µg/kg^−1^ in maize and by 67 µg/kg^−1^ in beans [2,85]. In multi-biofortification, a number of micronutrient traits are developed independently and combined in plants through backcrossing to obtain multi-biofortified varieties with many traits, such as beneficial micronutrients [32,104]. From the research findings by [85] on maize and beans in Kenya, a greater selenium increase was achieved during trials, making it highly recommended for cultivation under biofortification due to it being the main staple food in Malawi and other Southern African countries [71]. However, ref. [85] found that repeated applications of selenium are necessary to biofortify the crops each farming season, hence the need for fertilizer manufacturers to offer fertilizers that include selenium to farmers. Biofortification has been proven by scientists throughout the world to have beneficial effects on food crops grown across various climatic conditions and environments, resulting in positive outcomes on productive and economic agronomic traits [34,100,101,103]. Biofortification and stimulation were seen not only to enhance plant growth but also to aid in the reduction of the usage of chemical fertilizers and aid resistance by plants against various abiotic stresses and pathogens. According to [106], alfalfa biofortified with selenium fed to livestock daily is one of the most effective ways to improve selenium levels in animals, especially meat, which provides enough selenium to humans when consumed. Furthermore, it is important to note that concentrations of selenium in meat have been observed to be directly proportional to the selenium levels in feed and pastures, making biofortification a useful tool in increasing selenium levels for both humans and animals [2].

### 4.3. Animal Feed (Including Retention in Muscles and Liver, etc., for Human Consumption)

Research findings indicate that supplementation by selenium helps increase growth performance, the antioxidative ability of chickens, and selenium concentrations in meat and muscles [107]. Ref. [107] further observed that selenium fortification with yeast was more effective than selenium from sodium selenite in improving the meat quality of the broilers under study. In similar research, it was observed that selenium concentrations within human tissue mainly depend on the dietary intake of selenium, mainly linked to the selenium availability in the soil and its geographical distribution [97,108]. According to several research findings, measuring the selenium uptake in the diet of animals is a very difficult undertaking and is expensive [36,107]. Further studies indicate that the marker for adequate selenium intake by animals and humans is the measurement of GPx enzyme activity, which is measured in erythrocytes due to activities of this enzyme being directly proportional to dietary selenium intake [58,109]. In another research study in Korea and other Asian countries on pork and chicken eggs enriched with selenium, eggs are now being sold in 25 countries across the globe to help improve the selenium status of people after observing low levels of selenium in the population [3,69]. According to the safety tests conducted on biofortified rice by [102], no identifiable hazards for nutrients or other parameters indicated any adverse effects, making it very safe for animal consumption and a good tool for improving the selenium status in Southern Africa.

### 4.4. Supplements for Humans

Food biofortification may greatly benefit individuals with selenium deficiencies, but there is a great risk to people with a high, inherent selenium intake in their diet, who may be greatly affected by the biofortification process and outcomes [53]. Ref. [53] further recommended that people should not consume excessive amounts of foods fortified with selenium to avoid toxicity in those areas with high concentrations of selenium in the soil. Regarding selenium absorption in the body, retention of selenium in organic forms was found to be much higher than in inorganic forms [108]. Further findings by [108] on men using selenium yeast supplements concluded that selenium is well absorbed in the liver and retained in the human body, making it safe for uptake in required quantities. Research findings in Malawi indicated that the consumption of maize flour biofortified through selenium-enriched fertilizer addition to the soil resulted in an increased selenium status in the community under the study, providing strong proof of the principle that agronomic biofortification is an effective approach to address the selenium deficiency seen in Malawi and other African countries [78]. One good example of how biofortification was used to increase the selenium status of a population is Finland in 1960, where heart disease was the leading cause of death in the population due to low levels of selenium [55]. To control this problem, the Finnish government introduced selenium fertilizers in all kinds of fertilizer used for crops in 1984. The biofortification program was very successful by early 1990 in increasing plant concentrations of selenium, and levels of selenium in humans increased, leading to an improvement in the health status of the population. After observing all the benefits in the population, Finland adopted 6 mg Se/kg fertilizer as the application rate for all crops and later moved to 10 mg Se/kg [55,63]. Despite the public health measures done in Finland, [63] could not observe any positive linear trend for blood selenium over the years in a large laboratory database from 1987 to 2020 of Finnish people who were under study; this could be due to having outdated methods of analysis in the previous years. However, with proven success stories of countries like Finland, populations in Southern Africa can utilize biofortification as a tool that will improve the selenium levels and health status of its populations.

## 5. Summary of Research Findings on Selenium in Southern Africa

Based on the research conducted on selenium in Southern Africa, only a few countries have undertaken surveys and analyses of selenium levels for livestock and humans. There are serious research questions that need answers to properly map the selenium levels in Southern Africa. Below is a summary of the research findings conducted so far on soil, maize, the daily intake by humans, and human blood status.

**Figure 2:**
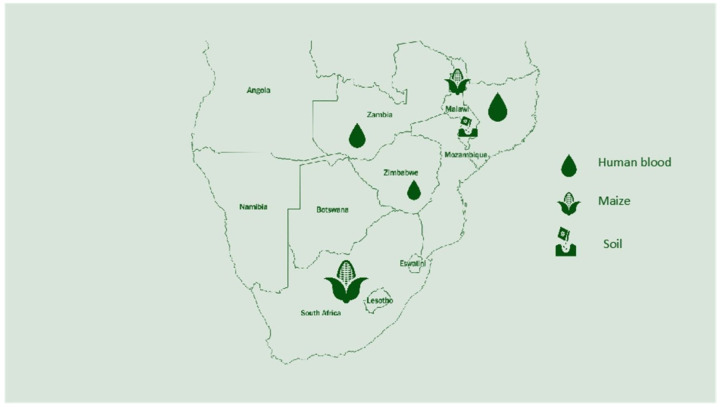
Research conducted on selenium in Southern Africa and areas studied.

The map below (Figure 2) shows the research that was conducted in each country to identify the selenium status in humans, soil, and crops. Overall, Malawi has conducted more research on selenium compared to other African countries. South Africa has conducted research on selenium but on specific topics that do not pinpoint the status of selenium in the country. Below are the countries with a known selenium status and analyzed parameters.

## 6. Conclusions and Future Outcomes

The literature and research findings strongly indicate some serious inadequacies of selenium in the soils of most parts of Southern African countries. Analyzed crop samples—mostly of maize, which is a staple food in most countries—proved to be deficient according to internationally acceptable livestock and human nutritional requirements. Maize grain in Southern Africa is, therefore, a poor source of selenium for humans and livestock feeding. Limited information on the selenium availability in grasses of Southern Africa is available, creating a research vacuum that requires further investigations. Further research outcomes of the analyzed soil samples by researchers strongly indicate limited amounts of selenium and require supplementation with selenium-rich fertilizers. Based on these studies, selenium supplementation can have many health benefits (e.g., improved production performance, growth, feed efficiency, antioxidant status, and immune status) when present in animal and human diets. Overall, the findings of this comprehensive review show that the consumption and levels of selenium are inadequate in Southern African nations and require intervention for the improved productivity and health of humans and livestock.
